# Ku80 is involved in telomere maintenance but dispensable for genomic stability in *Leishmania mexicana*

**DOI:** 10.1371/journal.pntd.0010041

**Published:** 2021-12-29

**Authors:** Ester Poláková, Amanda T. S. Albanaz, Alexandra Zakharova, Tatiana S. Novozhilova, Evgeny S. Gerasimov, Vyacheslav Yurchenko

**Affiliations:** 1 Life Science Research Centre, Faculty of Science, University of Ostrava, Ostrava, Czech Republic; 2 Faculty of Biology, M.V. Lomonosov Moscow State University, Moscow, Russia; 3 Institute for Information Transmission Problems, Russian Academy of Sciences, Moscow, Russia; 4 Martsinovsky Institute of Medical Parasitology, Tropical and Vector Borne Diseases, Sechenov University, Moscow, Russia; Vienna, AUSTRIA

## Abstract

**Background:**

Telomeres are indispensable for genome stability maintenance. They are maintained by the telomere-associated protein complex, which include Ku proteins and a telomerase among others. Here, we investigated a role of Ku80 in *Leishmania mexicana*. *Leishmania* is a genus of parasitic protists of the family Trypanosomatidae causing a vector-born disease called leishmaniasis.

**Methodology/Principal findings:**

We used the previously established CRISPR/Cas9 system to mediate ablation of Ku80- and Ku70-encoding genes in *L*. *mexicana*. Complete knock-outs of both genes were confirmed by Southern blotting, whole-genome Illumina sequencing, and RT-qPCR. Resulting telomeric phenotypes were subsequently investigated using Southern blotting detection of terminal restriction fragments. The genome integrity in the Ku80- deficient cells was further investigated by whole-genome sequencing.

Our work revealed that telomeres in the ΔKu80 *L*. *mexicana* are elongated compared to those of the wild type. This is a surprising finding considering that in another model trypanosomatid, *Trypanosoma brucei*, they are shortened upon ablation of the same gene. A telomere elongation phenotype has been documented in other species and associated with a presence of telomerase-independent alternative telomere lengthening pathway. Our results also showed that Ku80 appears to be not involved in genome stability maintenance in *L*. *mexicana*.

**Conclusion/Significance:**

Ablation of the Ku proteins in *L*. *mexicana* triggers telomere elongation, but does not have an adverse impact on genome integrity.

## Author summary

Parasites of the genus *Leishmania* cause leishmaniasis, a neglected tropical disease. Telomeric and sub-telomeric regions of these flagellates are often associated with virulence. That is why studying telomere maintenance is important. Here we demonstrate that deletion of one of the proteins, implicated in telomere maintenance, resulted in elongated telomeres. This is unexpected, because deletion of the same gene in another model organism, *Trypanosoma brucei*, has an opposite effect. Previous comparative genomic studies suggested that the protein under study might be also involved in genome stability. Nevertheless, we did not detect any trace of genomic instability in *L*. *mexicana* after 100 passages. We concluded that Ku80 is involved in telomere maintenance but dispensable for genomic stability in *Leishmania mexicana*.

## Introduction

*Leishmania* is a genus of parasitic protists of the family Trypanosomatidae [1,2]. It causes a vector-born disease called leishmaniasis, which affects vertebrates, including humans [3]. A total of 350 million people is at risk of infection with over 1 million new cases and 20,000–30,000 deaths being documented annually [4]. It is manifested in three different clinical forms–cutaneous, mucosal, and visceral. The majority of the visceral cases are fatal, if left untreated [5].

Ku70 and Ku80 are abundant and conserved DNA binding proteins remarkable for their versatile function [6]. They are best known for their essential role in maintaining genome integrity *via* non-homologous end joining (NHEJ) repair pathway, one of the two most commonly used pathways to repair double-stranded DNA breaks (DSBs) [7]. Being part of NHEJ, Ku proteins have far-reaching effects on such processes as V(D)J recombination and class switch recombination [8,9]. In NHEJ, the Ku protein complex is involved in the very initial step recognizing DSBs and binding there in the sequence-independent manner to protect it from the nucleolytic degradation. It later recruits a catalytic subunit of the DNA-dependent protein kinase and other proteins facilitating formation of the repair complex [10,11]. The NHEJ pathway is fairly conserved in all the studied species, yet there is a group of organisms that tend to lose it–parasites [12]. It is generally accepted that iconic parasites, trypanosomatids, do not employ NHEJ, but rather rely on sequence microhomology, to repair DSBs in the Ku heterodimer-independent manner [13]. This notion is further substantiated by the fact that ligase IV (an enzyme involved in the classical NHEJ) is absent from trypanosomatid genomes [14]. Nevertheless, both Ku70 and Ku80 are retained by virtually all (with just one exception) trypanosomatids, arguing that they may be used in other biochemical pathways. That sole exception is *Blastocrithidia* spp., which lack both Ku70 and Ku80 [15]. Notably, the genomes of these species have accumulated numerous insertions in protein-coding genes raising a question, whether such a level of genome instability may be associated with the loss of Ku proteins [12].

Apart from their indispensable role in DSB repair, the Ku proteins are also involved in other cellular processes, such as transcription, DNA damage response, DNA replication, and telomere maintenance, to name just a few [11,16]. At the chromosomal termini, they form complexes with other telomere-associated proteins protecting the DNA ends from being recognized and processed as DSBs [17] and preventing inappropriate recombination events involving telomeric repeats [18]. Surprisingly, the Ku proteins deficiency has manifested in rather ambiguous telomeric phenotypes in different species [19–24]. In a model trypanosomatid species, *Trypanosoma brucei*, complete ablation of the Ku80 protein resulted in gradual shortening of the telomeric repeats, a phenotype that was rescued by the expression of an ectopic copy of the gene [20]. The Ku protein knock-outs have been shown to influence the length of telomeres and trigger the alternative telomere lengthening (ALT) pathways that are not active under normal circumstances [25,26]. The canonical way of dealing with the “end replication problem” (an intrinsic inability of DNA polymerases to accomplish complete lagging strand synthesis of linear DNA templates leading to telomere shortening) [27,28] relies on engagement of a ribonucleoprotein enzyme, telomerase (TERT) [29]. The ALT pathways are TERT-independent and involve such molecular mechanisms as break-induced replication, replication using extrachromosomal DNA or a *t*-loop elongation [30,31]. Telomeres maintained by ALT are typically long and heterogeneous.

Telomeres of kinetoplastids possess the vertebrate canonical sequence (5′-ttaggg-3′), *t*-loops at their ends, and are maintained by TERT [32–34]. Of note, their genomes encode a conserved set of core proteins implicated in telomere maintenance [15,35].

In this paper, we show that ablation of the *LmxM*.*29*.*0340* (gene encoding Ku80 in *L*. *mexicana*) has resulted in the opposite telomeric phenotype, as compared to that in *T*. *brucei*. The telomers of *L*. *mexicana* get elongated upon deletion of Ku80. This phenotype was stable over at least 100 passages *in vitro* and was reversed back to the wild type by expression of the ectopic copy of Ku80. We also demonstrate that genome of *L*. *mexicana* ΔKu80 does not display instability traits, suggesting that Ku80 is not involved in its maintenance.

## Methods

### *Leishmania mexicana* axenic cultivation and analysis of growth kinetics

*Leishmania mexicana* (isolate MNYC/BZ/62/M379) promastigotes were grown in M199 medium supplemented with 2 μg/ml biopterin, 2 μg/ml hemin (all from Sigma-Aldrich, St. Louis, USA), 25 mM HEPES (Lonza, Basel, Switzerland), 50 units/ml of Penicillin/Streptomycin (Life Technologies/Thermo Fisher Scientific, Carlsbad, USA) and 10% heat-inactivated fetal bovine serum (BioSera Europe, Nuaillé, France) at 23°C.

Throughout the long-term experiment, WT and ΔKu80 cell cultures were passaged 2 times a week for a total of 100 passages. Cells were sampled from passage numbers 0, 25, 50, 75, and 100.

Growth kinetics *in vitro* was analyzed as described previously [36]. The statistical significance was evaluated using unpaired *t* test in Prism v. 8.0.1 (GraphPad Software, San Diego, USA).

### CRISPR-Cas9-mediated ablation of *LmxM*.*29*.*0340* (Ku80) and *LmxM*.*08_29*.*1050* (Ku70) in *Leishmania mexicana*

The CRISPR-Cas9 *L*. *mexicana* strain was established using the plasmid pTB007 [37]. To ablate *LmxM*.*29*.*0340* and *LmxM*.*08_29*.*1050*, guide RNAs (gRNAs) with a 20 nt seed sequence targeting upstream and downstream regions of the genes of interest were amplified using corresponding 5´gRNA and 3´gRNA primers (hereafter all primer sequences are listed in **S1 Table**). Donor DNA was PCR amplified from the pTNeo_v1 plasmid [38] with 5´ and 3´ primers, containing 30 nt homology regions, flanking corresponding gRNA targeting sites. The Cas9/T7 RNA polymerase-expressing promastigotes were cultivated in the M199 medium supplemented with 100 μg/ml Hygromycin B (VWR/ Avantor, Radnor, USA). The cells in mid-log phase were co-transfected with 5 μg of both 5´ and 3´gRNAs, along with 5 μg of donor DNA using the BTX ECM 630 electroporator (Harvard Apparatus Inc., Holliston, USA). Positive transfectants were selected on complete M199 medium supplemented with 100 μg/ml Hygromycin B and 50–100 μg/ml Neomycin G418 Sulfate (VWR/Avantor). Successful ablation of *LmxM*.*29*.*0340* was confirmed by the whole-genome sequencing (Illumina NovaSeq platform, Macrogen Europe B.V., Amsterdam, the Netherlands and the Institute of Applied Biotechnology, Prague, Czech Republic), RT-qPCR analysis, and Southern blotting. Successful ablation of *LmxM*.*08_29*.*1050* was confirmed by RT-qPCR. The resulting strains are hereafter referred to as *L*. *mexicana* ΔKu80 (*LmxM*.*29*.*0340*^-/-^) and ΔKu70 (*LmxM*.*08_29*.*1050*^-/-^).

For next-generation sequencing confirmation of complete ΔKu80 strain, the paired-end Illumina reads were trimmed with Trimmomatic v. 0.39 [39], mapped to the reference genome of *L*. *mexicana* isolate M379 [40] using Bowtie2 v. 2.3.5.1 [41], and the region corresponding to the gene of interest was visually inspected. The genome was also assembled *de novo* by SPAdes genome assembler v. 3.13.0 [42] and visualized with Tablet v. 1.19.09.03 [43].

To confirm complete *LmxM*.*29*.*0340* ablation in *L*. *mexicana* we also employed Southern blotting as previously described [44] using *Apa*I-digested total genomic DNA from the mid-log phase grown cells.

Finally, the expression of *LmxM*.*29*.*0340* and *LmxM*.*08_29*.*1050* was examined by RT-qPCR. Total RNA was isolated and transcript levels of the proteins of interest in *L*. *mexicana* were measured as described previously [45,46] in three biological and technical replicates. Expression values were normalized to those of the 18S rRNA.

### Ectopic expression of Ku80 in *L*. *mexicana* ΔKu80

The modified pLEXSY_IE-egfp-red-neo4 (Jena Bioscience GmbH, Jena, Germany) vector was used to express an ectopic copy of *LmxM*.*29*.*0340*. Firstly, the neomycin resistance gene of the pLEXSY_IE-egfp-red-neo4 was replaced by a gene encoding streptothricin acetyltransferase (*Sat*, allowing selection with Nourseothricin) to generate pLEXSY_IE-egfp-red-sat. The *Sat* ORF was amplified from the plasmid pLEXSY-Sat2 (Jena Bioscience) and cloned into the pLEXSY_IE-egfp-red-neo4 with *Bam*HI and *Spe*I (both from Thermo Fisher Scientific, Waltham, USA). The *LmxM*.*29*.*0340* ORF was amplified from the *L*. *mexicana* total genomic DNA and cloned into pLEXSY_IE-egfp-red-sat with *Bgl*II and *Not*I (Thermo Fisher Scientific), replacing EGFP-DsRed fusion gene. The resultant pLEXSY_IE-Ku80-sat plasmid was used for ectopic expression of *LmxM*.*29*.*0340*.

The *L*. *mexicana* ΔKu80 promastigotes were grown in the complete M199 medium supplemented with 100 μg/ml Hygromycin B and Neomycin G418 Sulfate. The mid-log phase cells were transfected with 5 μg of pLEXSY_IE-Ku80-sat plasmid as described above. Positive transfectants were selected on complete M199 medium supplemented with 100 μg/ml Hygromycin B and 50–100 μg/ml Nourseothricin (Jena Bioscience). Ectopic expression of *LmxM*.*29*.*0340* was confirmed by RT-qPCR and Southern blotting as described above. The resulting strain is hereafter referred to as *L*. *mexicana* Ku80_add.

### *De novo* sequencing and genome assembly

*Leishmania mexicana* (isolate MNYC/BZ/62/M379) genome was also assembled *de novo* with Flye v. 2.8.3 [47] using only PacBio reads reported previously [48] and genome size parameter 32 Mb, which is the size of reference *L*. *mexicana* MHOM/GT/2001/U1103 assembly [49] from the TriTrypDB release 52 [50]. This has been done in order to assemble longer contigs needed for genome instability analyses. The PacBio read N_50_ and average genome coverage estimated by Flye were 9,022 bp and 130×, respectively. The initial Flye assembly with contig N_50_ of 1,043 kbp and 73 total number of fragments was polished twice (Racon polishing) with TGS-GapCloser v. 1.1.1 [51], and then with Pilon v. 1.23 [52] using 30 million of high-quality Illumina read pairs (read length 150 bp). At this stage, the assembled scaffolds were broken into contigs, which were re-scaffolded with RaGOO v. 1.1 [53] using TriTrypDB *L*. *mexicana* assembly as a reference. The resulting assembly was again treated with TGS-GapCloser and no genome rearrangements (relative to *L*. *mexicana* MHOM/GT/2001/U1103) were detected at this step. The final assembly was quality-checked using QUAST v. 5.0.2 [54] and BUSCO v. 5.0.0 [55] tools.

### Genome instability analysis

*Leishmania mexicana* genomes (ΔKu80 and wild type: 0, 25, 50, 75, 100 passages; 10 samples in total) were sequenced using Illumina NovaSeq platform at the Institute of Applied Biotechnology. Sequencing reads data obtained in frame of this work are deposed into NCBI SRA under BioProject PRJNA746247. Random subsamples of ~17–18 million of read pairs were prepared with a custom Python script from each sequenced sample after their quality check and adapter trimming with Trimmomatic v. 0.39 [39]. Each subsample was mapped on the genome assembly using the BWA mem v. 0.7.17 [56] and alignments were processed with SAMtools v. 1.9 [57]. The SNP and short indel calling was performed with the Genome Analysis Toolkit (GATK) v. 4.2 using the ‘HaplotypeCaller’ tool [58] with default settings and with ‘-ploidy’ set to 10 to capture possible variants with low frequency, as possible SNPs or indels caused by the Ku80 ablation could potentially be restricted to a fraction of cells only. The SNP and indel sets were compared using a custom Python script. Chimeric and split alignments, secondary alignments and read alignments with soft-clipped bases were counted in SAM files with custom shell scripts, using specific SAM tags and alignment CIGAR strings. General read mapping analyses were focused on counting the fractions of reads with specific properties, which can point to various recombination or mutation processes in genomes: number of unmapped, soft-clipped, chimeric alignments. SA:Z and XA:Z tags were used by BWA short read aligner for chimeric/split read alignments. Each subsample’s sorted BAM file was analyzed for assembly identity using the ALE tool [59].

### Plotting chimeric reads density

As a control dataset, a sample of *L*. *mexicana* M379, sequenced by The Wellcome Trust Sanger Institute in 2013 and deposed in NCBI SRA under accession ERR307335, was downloaded and processed as above. Reads were mapped on the M379 genome assembly with BWA mem v. 0.7.17. Chromosome sequence was binned in 24 kbps-long fragments, bin number was assigned to each mapped read or read fragment. Read counts were stored in 2D-matrix in such a way that the row in this matrix corresponds to the bin number, assigned to the first read in pair (or the ‘leftmost’ mapped read fragment) and the column is the bin number assigned to the second read in read pair (or ‘rightmost’ mapped read fragment). The read pairs mapped close to each other were counted in cells near diagonal of such matrix, while chimeric reads of putative “translocations” were counted in cells far from diagonal. Read counts in the matrix were first normalized over the total number of mapped reads, then diagonal elements of the matrices were replaced with zeros and all other values were scaled to (0, 1) range. As the entries of matrices are symmetrical across main diagonal, matrices for two different samples were joined.

### Southern blotting and quantification of transcripts encoding telomere-associated proteins using RT-qPCR

Telomere lengths were analyzed by the terminal restriction fragment length analysis as in [15,20]. For the loading and integrity control, the membranes were stripped and re-probed against a fragment of an 18S rRNA gene. Statistics of the telomere lengths were obtained with an online tool WALTER (Web-based Analyser of the Length of TElomeRes) [60].

Transcripts encoding telomere-associated proteins were quantified by RT-qPCR in five biological replicates as in [15].

## Results

### Establishment of the ΔKu80 (*LmxM*.*29*.*0340*^-/-^) and add-back *L*. *mexicana* lines

**Fig 1:**
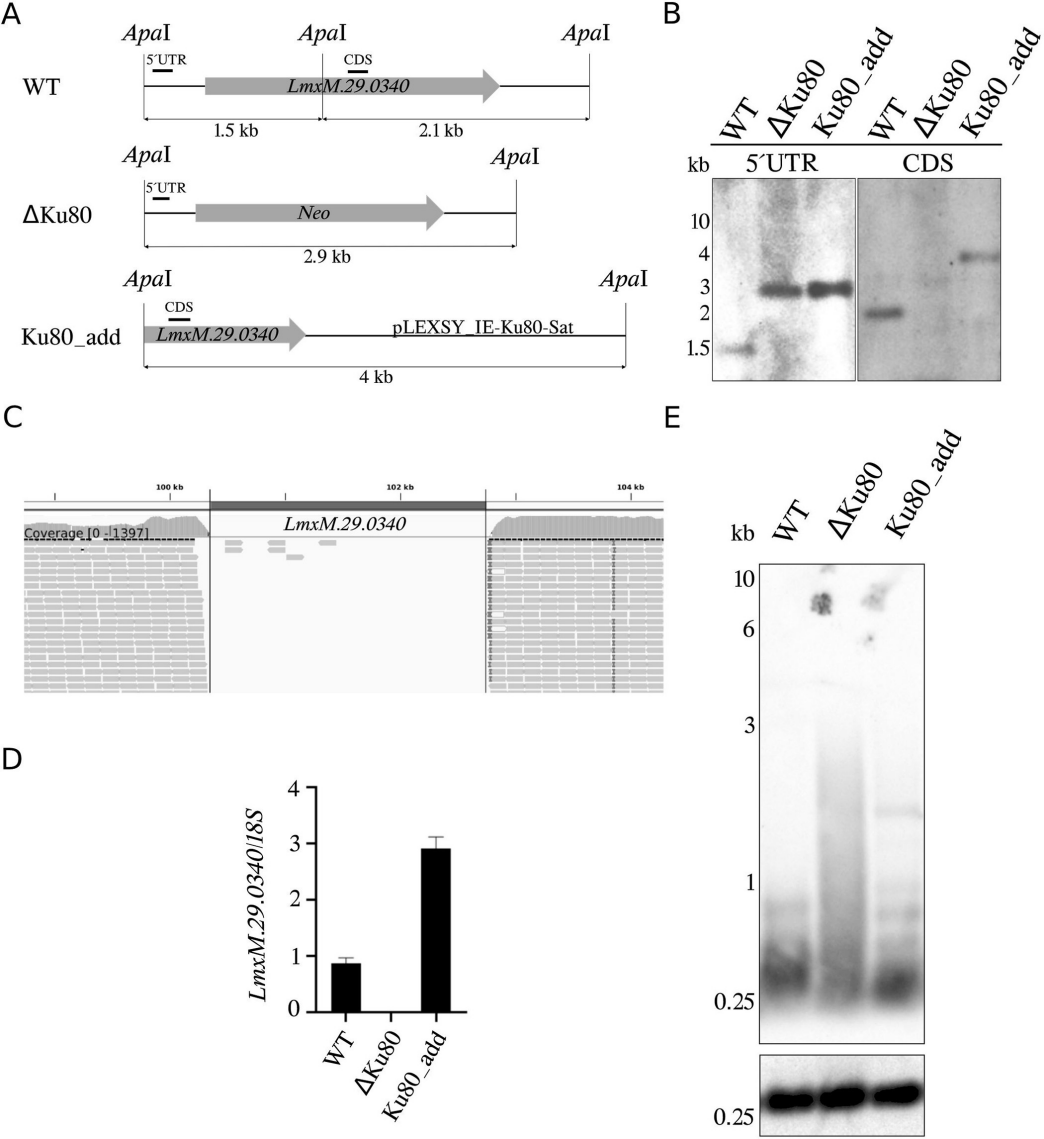
Establishment of the ΔKu80 (*LmxM*.*29*.*0340*^-/-^) and add-back *L*. *mexicana* and analysis of telomeres in these lines. **A**) Strategy for *LmxM*.*29*.*0340* ablation and add-back confirmation by Southern blotting. Annealing positions of probes and expected fragment sizes are shown. **B**) Southern blotting analysis of the *Apa*I digested *L*. *mexicana* genomic DNA of the WT, ΔKu80 and Ku80_add strains with 5´UTR and CDS probes. **C**) Illumina sequencing reads mapping to the *LmxM*.*29*.*0340* locus of chromosome 29 in *L*. *mexicana* ΔKu80 genome. **D**) Quantitative RT–PCR analysis of *LmxM*.*29*.*0340* expression in WT, ΔKu80 and Ku80_add *L*. *mexicana* strains. **E**) Southern blotting analysis of the telomere repeats in WT, ΔKu80 and Ku80_add *L*. *mexicana* strains. Membranes were hybridized with a telomeric probe (upper panel) and a probe against 18S gene (lower panel, DNA integrity control). Sizes of DNA fragments in kb (GeneRuler 1 kb DNA ladder, Thermo Fisher Scientific) are indicated on the left in B and E.

In order to investigate function of the Ku proteins in *Leishmania* biology, we first ablated the Ku80-encoding gene (*LmxM*.*29*.*0340*) in *L*. *mexicana*. The clonal cultures, deficient in both *LmxM*.*29*.*0340* alleles (*L*. *mexicana* ΔKu80), were obtained using CRISPR/Cas9 system [38] (**Fig 1A**). The complete knock-out was confirmed by Southern blotting (**Fig 1B**), whole-genome Illumina sequencing (**Fig 1C**), and RT–qPCR (**Fig 1D**). In addition, we also established the add-back cultures, in which Ku80 was overexpressed episomally on the *LmxM*.*29*.*0340*-null background (**Fig 1A**) and verified it by Southern blotting (**Fig 1B**) and RT–qPCR (**Fig 1D**). Of note, the expression level of *LmxM*.*29*.*0340* in the add-back cells was higher compared to that of the wild type, but these numbers were not as dramatically different as often observed in the add-back experiments [61,62].

To exclude the clonal bias in data interpretation, we have selected four and three random clones of ΔKu80 and Ku80_add *L*. *mexicana*, respectively. As an additional control, we have also established a clonal line with ablated gene *LmxM*.*08_29*.*1050* encoding Ku70 (**S1 Fig**).

### Telomeres in *L*. *mexicana* ΔKu80 are elongated and this phenotype is stable over 100 passages

*Leishmania mexicana* is a model species with extremely short telomeres, as compared to other trypanosomatids [15]. Contrary to our expectations (based on the previously reported studies from another model trypanosomatid *T*. *brucei* [20]), the *L*. *mexicana* ΔKu80 telomeres were considerably extended upon *LmxM*.*29*.*0340* ablation (median telomere length 389 and 564 bp for the wild type and ΔKu80 cells, respectively). This phenotype was reversed in the add-back cells (median telomere length 431 bp), confirming that the observed effect is not an artefact of the genetic manipulations (**Fig 1E** and **S2 Table**). Of note, the results were similar in all analyzed randomly selected ΔKu80 and add-back clonal lines. Remarkably, the ablation of another Ku protein, Ku70, also manifested in elongated telomeres (**S2 Fig** and **S2 Table**).

**Fig 2:**
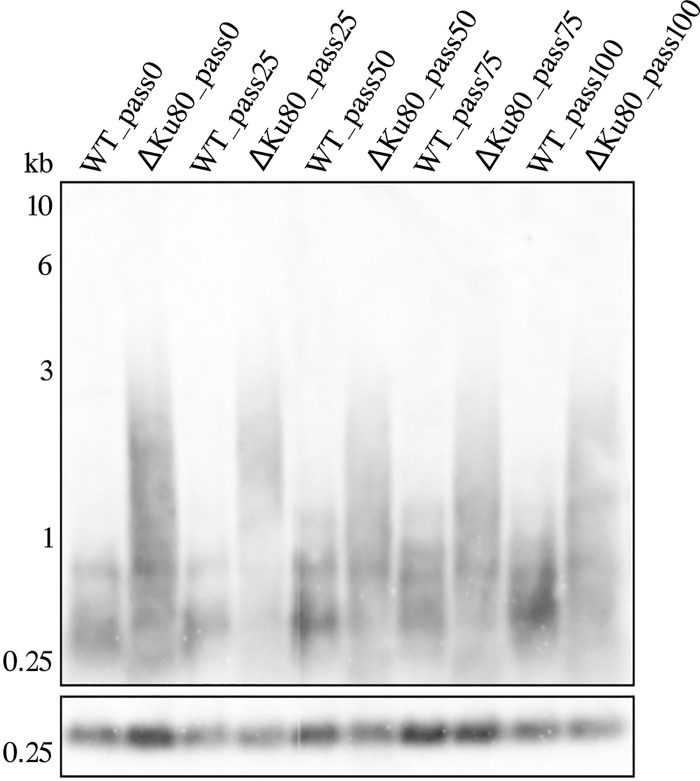
Elongated telomers in *L*. *mexicana* ΔKu80 are stable over 100 passages *in vitro*. Southern blotting analysis of the telomere repeats in WT, ΔKu80 *L*. *mexicana* strains passaged for 0, 25, 50, 75, and 100 passages. Membranes were hybridized with a telomeric probe (upper panel) and a probe against 18S gene (lower panel, DNA integrity control). Sizes of DNA fragments in kb (GeneRuler 1 kb DNA ladder) are indicated on the left.

We also investigated whether the observed telomeric phenotype is stable and confirmed that it is not altered for over 100 passages in culture (**Fig 2**). The *L*. *mexicana* cells with ablated Ku80 divide slightly slower compared to their wild type counterparts; this effect is reversed in the add-back lines (**S3 Fig**).

### Expression patterns of some telomere–associated proteins and Ku80 correlate in *L*. *mexicana*

**Fig 3:**
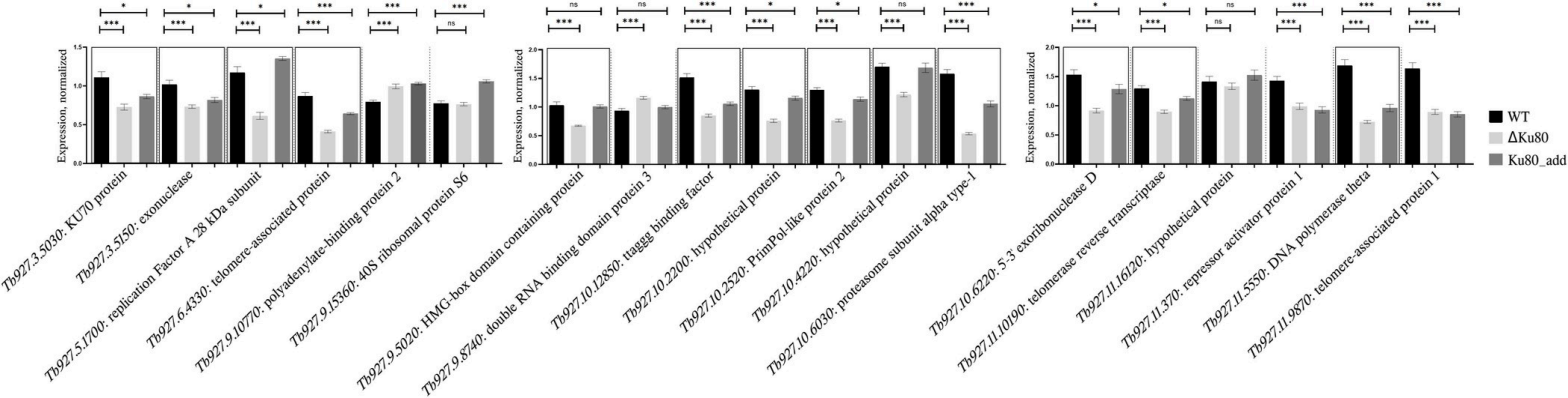
Transcript levels of telomere-associated proteins in WT, ΔKu80, and Ku80_add strains of *L*. *mexicana*. Gene expression is presented as normalized means with SEM derived from five independent biological replicates. *T*. *brucei* IDs of telomeric proteins are used for consistency with previously published results. Data for transcripts, whose expression pattern correlates with that of Ku80, are boxed. Statistical significance is indicated as follows: * < 0.05; ** <0.01; *** <0.001; ns–not significant.

We have recently shown that the majority of proteins associated with telomeres in *T*. *brucei* are conserved across Trypanosomatidae [15]. To investigate the potential correlation between expression of the *LmxM*.*29*.*0340* and other genes encoding proteins implicated in telomere maintenance in trypanosomatids [15,35], we analyzed their transcription level in the WT, ΔKu80, and Ku80_add *L*. *mexicana* cells. Our results show that expression pattern (at the level of RNA) of most of these proteins (13 out of 19) correlated with that of *LmxM*.*29*.*0340* (**Fig 3**, boxed). Compared to the wild type, their expression was statistically significant down and up regulated in the ΔKu80 and Ku80_add *L*. *mexicana*, respectively. Some notable entries on this list are TERT, ttaggg binding factor (TRF), and repressor activator protein 1 (RAP1), which confirm previous observations of their interactions with Ku proteins in other species [21,22,25,63–65]. This is not a genome-wide phenomenon, as some of the analyzed genes do not follow the same pattern (**Fig 3**).

### Deeper sequencing and genome instability analyses

In order to investigate possible influence of the Ku80 ablation on genome stability, we first performed high-quality chromosome-level genome assembly of *L*. *mexicana* MNYC/BZ/62/M379 (hereafter referred as “M379 assembly”) and compared it to that of the *L*. *mexicana* MHOM/GT/2001/U1103 assembly from the TriTrypDB (“reference assembly”). The final M379 assembly has 34 nuclear chromosomes (31,72 Mbps in total) along with a completely assembled circular contig of the mitochondrial maxicircle. The QUAST report indicated that 97.8% of the Illumina reads were mapped back onto the assembly, 99.99% of bases had non-zero coverage, and 99.98% of the reference *L*. *mexicana* assembly was covered by the M379 assembly contigs. The M379 assembly had averaged 10 Ns per 100 kb. In total, 594 sequence discrepancies were detected between the M379 and the reference *L*. *mexicana* assemblies. Most of these variants are rather short (under 1 kb) insertions or deletions (400 cases) or tandem repeat expansions/contractions (96 cases); both types can be frequently observed in trypanosomatid genomes. No miss-assemblies were detected by Pilon, TGS-GapCloser, or read mapping of either long PacBio or paired-end Illumina reads. The BUSCO completeness of the M379 assembly for ‘eukaryota’ dataset is 53.8% (137 out of 255 complete BUSCOs). This is the value comparable to the best available chromosome-level assemblies for trypanosomatids (*Leishmania tarentolae*: 53.8%, *L*. *mexicana*: 53.8%, *L*. *major*: 53.8%, *Leptomonas pyrrhocoris*: 55.3%, *T*. *brucei*: 55.7%). Altogether, this indicates that vast majority of the *L*. *mexicana* MNYC/BZ/62/M379 genome was properly assembled.

**Fig 4:**
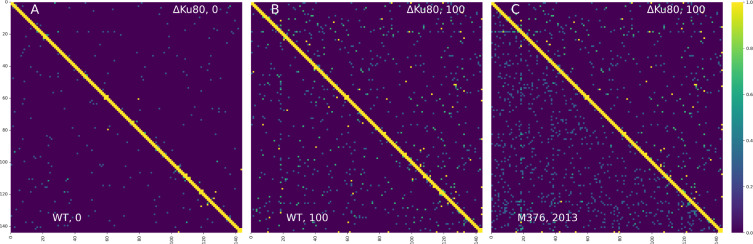
Chimeric reads density for the 20^th^ chromosome of different *Leishmania mexicana* strains. ΔKu80 and WT are samples from the current study, shown at the beginning of cultivation experiment (**A**) and on the 100^th^ passage (**B**). Panel **C**) compares the whole-genome dataset sequenced by The Wellcome Trust Sanger Institute in 2013 (labeled M379, 2013) to the ΔKu80 strain after 100 passages. Normalized read density is plotted.

Next, we investigated genome stability by mapping the paired-end Illumina reads (150 bp) obtained from the WT and ΔKu80 *L*. *mexicana* collected after 0, 25, 50, 75 and 100 passages *in vitro*. Read mappings were analyzed with various tools that detect SNPs, short indels, longer structural variants indicated by the chimeric read alignments or misaligned read pairs, locus coverage variations, and overall likelihood of the assembly (ALE method). As genomic variations, caused by malfunction of the DNA repair system, can arise randomly in cells, the ‘allele frequency’ of each individual variant can be rather low. We accounted for that in the GATK haplotype calling by adjusting the ploidy parameter. We also analyzed the possible variants present at extremely low frequencies by counting overall number of mis-aligned, chimeric or soft-clipped reads in each sample (these read alignments indicate the sequence difference between the read and the reference and can capture events supported even by a single read sequence). The Wilcoxon signed rank test was used to conduct pairwise comparisons between the WT and ΔKu80 samples and no differences were found in any test (*p*-value < 0.05). Results and statistics of the analyses are summarized in the **S3 Table**. This is illustrated in the **Fig 4** for chromosome 20 of *L*. *mexicana*. In both cell lines (ΔKu80 and WT), the observed frequency of putative translocations slightly increases over time (compare panels A and B), but this increasement is similar. Notably, we detected a higher translocation frequency when we compared genomic data for the same strain produced in 2013 and 2021 (panel C). This can be easily explained by the plasticity of *Leishmania* genomes–recombination events accumulate over time [66–68].

To conclude, we did not detect any statistically significant difference between the WT and ΔKu80 samples in any performed test. These results imply that ablation of *LmxM*.*29*.*0340* does not result in genome instability; although they do not exclude a possibility that the effect of this knock-out is delayed and manifests itself after more rounds of cell division.

## Discussion

In this work we investigated the role of Ku80 in *Leishmania mexicana* biology and demonstrated that its ablation affects telomere length. Unlike situation in another model trypanosomatid species, *Trypanosoma brucei* [20], a complete knock-out of the Ku80 encoding gene (*LmxM*.*29*.*0340*) resulted in telomere elongation (**Fig 1**). Of note, and, again, different from *T*. *brucei*, elongated telomeres in *L*. *mexicana* are stable for at least 100 passages in culture (**Fig 2**). A similar phenotype (telomere elongation upon Ku protein ablation) was previously observed in *C*. *albicans* and *A*. *thaliana* [21,22]. In these species, telomere elongation in the Ku deficient mutants was linked to alternative TERT-independent ways of telomere maintaining [21,25,69]. Notably, alternative mechanisms of telomer maintenance have been recently documented in *Leishmania* spp. [70]. The Ku 80 protein in *L*. *mexicana* appears to be central for the network of factors involved in telomere maintenance, as its expression at RNA level correlates with that of 13 out of 19 genes previously implicated in this process (**Fig 3**). On the other hand, we did not corroborate a recently proposed hypothesis that Ku proteins might direct genome stability in trypanosomatids [12].

## Supporting information

S1 Table Primers used in this study. (XLSX)

S2 Table Telomere lengths (weighted median, minimum–maximum) in analyzed clonal lines of *L*. *mexicana*. (XLSX)

S3 Table Genome instability analyses summary. (XLSX)

S1 Fig Establishment of the ΔKu70 *L*. *mexicana*. **A**) Strategy for *LmxM*.*08_29*.*1050* ablation. **B**) Quantitative RT–PCR analysis of *LmxM*.*08_29*.*1050* expression in WT and ΔKu70 *L*. *mexicana* strains.
(JPG)

S2 Fig Southern blotting analysis of the telomere repeats in WT, ΔKu70 (1 clonal line), ΔKu80 (4 clonal lines) and Ku80_add (3 clonal lines) *L*. *mexicana* strains. Sizes of DNA fragments in kb are indicated on the left. (JPG)

S3 Fig Growth curves of the wild type, ΔKu80 (3 clonal lines) and Ku80_add (3 clonal lines) *L*. *mexicana* strains. Statistical significance: ** <0.01. (JPG)

## References

[1] Kostygov AY, Karnkowska A, Votýpka J, Tashyreva D, Maciszewski K, Yurchenko V, et al. Euglenozoa: taxonomy, diversity and ecology, symbioses and viruses. Open Biol. 2021;11: 200407. doi: 10.1098/rsob.200407 33715388 PMC8061765

[2] Maslov DA, Opperdoes FR, Kostygov AY, Hashimi H, Lukeš J, Yurchenko V. Recent advances in trypanosomatid research: genome organization, expression, metabolism, taxonomy and evolution. Parasitology. 2019;146: 1–27. doi: 10.1017/S0031182018000951 29898792

[3] Bruschi F, Gradoni L. The leishmaniases: old neglected tropical diseases. Springer. 2018.

[4] WHO. Leishmaniasis. 2020 (Cited November 19 2021). Available from: https://www.who.int/en/news-room/fact-sheets/detail/leishmaniasis

[5] Stuart K, Brun R, Croft S, Fairlamb A, Gurtler RE, McKerrow J, et al. Kinetoplastids: related protozoan pathogens, different diseases. J Clin Invest. 2008;118: 1301–1310. doi: 10.1172/JCI33945 18382742 PMC2276762

[6] Aravind L, Koonin EV. Prokaryotic homologs of the eukaryotic DNA-end-binding protein Ku, novel domains in the Ku protein and prediction of a prokaryotic double-strand break repair system. Genome Res. 2001;11: 1365–1374. doi: 10.1101/gr.181001 11483577 PMC311082

[7] Chang HHY, Pannunzio NR, Adachi N, Lieber MR. Non-homologous DNA end joining and alternative pathways to double-strand break repair. Nat Rev Mol Cell Biol. 2017;18: 495–506. doi: 10.1038/nrm.2017.48 28512351 PMC7062608

[8] Williams GJ, Hammel M, Radhakrishnan SK, Ramsden D, Lees-Miller SP, Tainer JA. Structural insights into NHEJ: building up an integrated picture of the dynamic DSB repair super complex, one component and interaction at a time. DNA Repair. 2014;17: 110–120. doi: 10.1016/j.dnarep.2014.02.009 24656613 PMC4102006

[9] Zan H, Tat C, Qiu Z, Taylor JR, Guerrero JA, Shen T, et al. Rad52 competes with Ku70/Ku86 for binding to S-region DSB ends to modulate antibody class-switch DNA recombination. Nat Commun. 2017;8: 14244. doi: 10.1038/ncomms14244 28176781 PMC5309807

[10] Abbasi S, Schild-Poulter C. Mapping the Ku interactome using proximity-dependent biotin identification in human cells. J Proteome Res. 2019;18: 1064–1077. doi: 10.1021/acs.jproteome.8b00771 30585729

[11] Fell VL, Schild-Poulter C. The Ku heterodimer: function in DNA repair and beyond. Mutat Res Rev Mutat Res. 2015;763: 15–29. doi: 10.1016/j.mrrev.2014.06.002 25795113

[12] Nenarokova A, Záhonová K, Krasilnikova M, Gahura O, McCulloch R, Ziková A, et al. Causes and effects of loss of classical nonhomologous end joining pathway in parasitic eukaryotes. mBio. 2019;10: e01541–01519. doi: 10.1128/mBio.01541-19 31311886 PMC6635534

[13] Burton P, McBride DJ, Wilkes JM, Barry JD, McCulloch R. Ku heterodimer-independent end joining in *Trypanosoma brucei* cell extracts relies upon sequence microhomology. Eukaryot Cell. 2007;6: 1773–1781. doi: 10.1128/EC.00212-07 17693593 PMC2043400

[14] Genois MM, Paquet ER, Laffitte MC, Maity R, Rodrigue A, Ouellette M, et al. DNA repair pathways in trypanosomatids: from DNA repair to drug resistance. Microbiol Mol Biol Rev. 2014;78: 40–73. doi: 10.1128/MMBR.00045-13 24600040 PMC3957735

[15] Poláková E, Záhonová K, Albanaz ATS, Butenko A, Lukeš J, Yurchenko V. Diverse telomeres in trypanosomatids. Parasitology. 2021;148: 1254–1270. doi: 10.1017/S0031182021000378 33612129 PMC8311970

[16] Abbasi S, Parmar G, Kelly RD, Balasuriya N, Schild-Poulter C. The Ku complex: recent advances and emerging roles outside of non-homologous end-joining. Cell Mol Life Sci. 2021;78: 4589–4613. doi: 10.1007/s00018-021-03801-1 33855626 PMC11071882

[17] Indiviglio SM, Bertuch AA. Ku’s essential role in keeping telomeres intact. Proc Natl Acad Sci U S A. 2009;106: 12217–12218. doi: 10.1073/pnas.0906427106 19622731 PMC2718394

[18] Fisher TS, Zakian VA. Ku: a multifunctional protein involved in telomere maintenance. DNA Repair. 2005;4: 1215–1226. doi: 10.1016/j.dnarep.2005.04.021 15979949

[19] Boulton SJ, Jackson SP. Components of the Ku-dependent non-homologous end-joining pathway are involved in telomeric length maintenance and telomeric silencing. EMBO J. 1998;17: 1819–1828. doi: 10.1093/emboj/17.6.1819 9501103 PMC1170529

[20] Janzen CJ, Lander F, Dreesen O, Cross GA. Telomere length regulation and transcriptional silencing in Ku80-deficient *Trypanosoma brucei*. Nucleic Acids Res. 2004;32: 6575–6584. doi: 10.1093/nar/gkh991 15602000 PMC545459

[21] Chico L, Ciudad T, Hsu M, Lue NF, Larriba G. The *Candida albicans* Ku70 modulates telomere length and structure by regulating both telomerase and recombination. PLoS One. 2011;6: e23732. doi: 10.1371/journal.pone.0023732 21886818 PMC3160324

[22] Riha K, Shippen DE. Ku is required for telomeric C-rich strand maintenance but not for end-to-end chromosome fusions in *Arabidopsis*. Proc Natl Acad Sci U S A. 2003;100: 611–615. doi: 10.1073/pnas.0236128100 12511598 PMC141044

[23] Espejel S, Franco S, Rodriguez-Perales S, Bouffler SD, Cigudosa JC, Blasco MA. Mammalian Ku86 mediates chromosomal fusions and apoptosis caused by critically short telomeres. EMBO J. 2002;21: 2207–2219. doi: 10.1093/emboj/21.9.2207 11980718 PMC125978

[24] Samper E, Goytisolo FA, Slijepcevic P, van Buul PP, Blasco MA. Mammalian Ku86 protein prevents telomeric fusions independently of the length of TTAGGG repeats and the G-strand overhang. EMBO Rep. 2000;1: 244–252. doi: 10.1093/embo-reports/kvd051 11256607 PMC1083725

[25] Zellinger B, Akimcheva S, Puizina J, Schirato M, Riha K. Ku suppresses formation of telomeric circles and alternative telomere lengthening in *Arabidopsis*. Mol Cell. 2007;27: 163–169. doi: 10.1016/j.molcel.2007.05.025 17612498

[26] Sui J, Zhang S, Chen BPC. DNA-dependent protein kinase in telomere maintenance and protection. Cell Mol Biol Lett. 2020;25: 2. doi: 10.1186/s11658-020-0199-0 31988640 PMC6969447

[27] Olovnikov AM. A theory of marginotomy. The incomplete copying of template margin in enzymic synthesis of polynucleotides and biological significance of the phenomenon. J Theor Biol. 1973;41: 181–190. doi: 10.1016/0022-5193(73)90198-7 4754905

[28] Greider CW. Telomere length regulation. Annu Rev Biochem. 1996;65: 337–365. doi: 10.1146/annurev.bi.65.070196.002005 8811183

[29] Greider CW. Telomeres, telomerase and senescence. Bioessays. 1990;12: 363–369. doi: 10.1002/bies.950120803 2241933

[30] Lundblad V. Telomere maintenance without telomerase. Oncogene. 2002;21: 522–531. doi: 10.1038/sj.onc.1205079 11850777

[31] McEachern MJ, Blackburn EH. Cap-prevented recombination between terminal telomeric repeat arrays (telomere CPR) maintains telomeres in *Kluyveromyces lactis* lacking telomerase. Genes Dev. 1996;10: 1822–1834. doi: 10.1101/gad.10.14.1822 8698241

[32] Muñoz-Jordán JL, Cross GA. Telomere shortening and cell cycle arrest in *Trypanosoma brucei* expressing human telomeric repeat factor TRF1. Mol Biochem Parasitol. 2001;114: 169–181. doi: 10.1016/s0166-6851(01)00259-6 11378197

[33] Conte FF, Cano MI. Genomic organization of telomeric and subtelomeric sequences of *Leishmania* (*Leishmania*) *amazonensis*. Int J Parasitol. 2005;35: 1435–1443. doi: 10.1016/j.ijpara.2005.05.011 16126212

[34] Fulnečková J, Ševčíková T, Fajkus J, Lukešová A, Lukeš M, Vlček Č, et al. A broad phylogenetic survey unveils the diversity and evolution of telomeres in eukaryotes. Genome Biol Evol. 2013;5: 468–483. doi: 10.1093/gbe/evt019 23395982 PMC3622300

[35] Reis H, Schwebs M, Dietz S, Janzen CJ, Butter F. TelAP1 links telomere complexes with developmental expression site silencing in African trypanosomes. Nucleic Acids Res. 2018;46: 2820–2833. doi: 10.1093/nar/gky028 29385523 PMC5888660

[36] Ishemgulova A, Kraeva N, Hlaváčová J, Zimmer SL, Butenko A, Podešvová L, et al. A putative ATP/GTP binding protein affects *Leishmania mexicana* growth in insect vectors and vertebrate hosts. PLoS Negl Trop Dis. 2017;11: e0005782. doi: 10.1371/journal.pntd.0005782 28742133 PMC5542692

[37] Beneke T, Madden R, Makin L, Valli J, Sunter J, Gluenz E. A CRISPR Cas9 high-throughput genome editing toolkit for kinetoplastids. R Soc Open Sci. 2017;4: 170095. doi: 10.1098/rsos.170095 28573017 PMC5451818

[38] Beneke T, Gluenz E. LeishGEdit: a method for rapid gene knockout and tagging using CRISPR-Cas9. In: Clos J, editor. *Leishmania*. New York, NY: Humana Press; 2019. pp. 189–210. 10.1007/978-1-4939-9210-2_9 30980304

[39] Bolger AM, Lohse M, Usadel B. Trimmomatic: a flexible trimmer for Illumina sequence data. Bioinformatics. 2014;30: 2114–2120. doi: 10.1093/bioinformatics/btu170 24695404 PMC4103590

[40] Ishemgulova A, Hlaváčová J, Majerová K, Butenko A, Lukeš J, Votýpka J, et al. CRISPR/Cas9 in *Leishmania mexicana*: a case study of LmxBTN1. PLoS One. 2018;13: e0192723. doi: 10.1371/journal.pone.0192723 29438445 PMC5811015

[41] Langmead B, Salzberg SL. Fast gapped-read alignment with Bowtie 2. Nat Methods. 2012;9: 357–359. doi: 10.1038/nmeth.1923 22388286 PMC3322381

[42] Bankevich A, Nurk S, Antipov D, Gurevich AA, Dvorkin M, Kulikov AS, et al. SPAdes: a new genome assembly algorithm and its applications to single-cell sequencing. J Comput Biol. 2012;19: 455–477. doi: 10.1089/cmb.2012.0021 22506599 PMC3342519

[43] Milne I, Bayer M, Stephen G, Cardle L, Marshall D. Tablet: visualizing next-generation sequence assemblies and mappings. Methods Mol Biol. 2016;1374: 253–268. doi: 10.1007/978-1-4939-3167-5_14 26519411

[44] Kraeva N, Leštinová T, Ishemgulova A, Majerová K, Butenko A, Vaselek S, et al. *LmxM.22.0250*-encoded dual specificity protein/lipid phosphatase impairs *Leishmania mexicana* virulence *in vitro*. Pathogens. 2019;8: 241. doi: 10.3390/pathogens8040241 31744234 PMC6969907

[45] Záhonová K, Hadariová L, Vacula R, Yurchenko V, Eliáš M, Krajčovič J, et al. A small portion of plastid transcripts is polyadenylated in the flagellate *Euglena gracilis*. FEBS Lett. 2014;588: 783–788. doi: 10.1016/j.febslet.2014.01.034 24492004

[46] Ishemgulova A, Kraeva N, Faktorová D, Podešvová L, Lukeš J, Yurchenko V. T7 polymerase-driven transcription is downregulated in metacyclic promastigotes and amastigotes of *Leishmania mexicana*. Folia Parasitol. 2016;63: 016. doi: 10.14411/fp.2016.016 27311571

[47] Kolmogorov M, Yuan J, Lin Y, Pevzner PA. Assembly of long, error-prone reads using repeat graphs. Nat Biotechnol. 2019;37: 540–546. doi: 10.1038/s41587-019-0072-8 30936562

[48] Sádlová J, Podešvová L, Bečvář T, Bianchi C, Gerasimov ES, Saura A, et al. Catalase impairs *Leishmania mexicana* development and virulence. Virulence. 2021;12: 852–867. doi: 10.1080/21505594.2021.1896830 33724149 PMC7971327

[49] Rogers MB, Hilley JD, Dickens NJ, Wilkes J, Bates PA, Depledge DP, et al. Chromosome and gene copy number variation allow major structural change between species and strains of *Leishmania*. Genome Res. 2011;21: 2129–2142. doi: 10.1101/gr.122945.111 22038252 PMC3227102

[50] Aslett M, Aurrecoechea C, Berriman M, Brestelli J, Brunk BP, Carrington M, et al. TriTrypDB: a functional genomic resource for the Trypanosomatidae. Nucleic Acids Res. 2010;38: D457–D462. doi: 10.1093/nar/gkp851 19843604 PMC2808979

[51] Xu M, Guo L, Gu S, Wang O, Zhang R, Peters BA, et al. TGS-GapCloser: a fast and accurate gap closer for large genomes with low coverage of error-prone long reads. Gigascience. 2020;9: giaa094. doi: 10.1093/gigascience/giaa094 32893860 PMC7476103

[52] Walker BJ, Abeel T, Shea T, Priest M, Abouelliel A, Sakthikumar S, et al. Pilon: an integrated tool for comprehensive microbial variant detection and genome assembly improvement. PLoS One. 2014;9: e112963. doi: 10.1371/journal.pone.0112963 25409509 PMC4237348

[53] Alonge M, Soyk S, Ramakrishnan S, Wang X, Goodwin S, Sedlazeck FJ, et al. RaGOO: fast and accurate reference-guided scaffolding of draft genomes. Genome Biol. 2019;20: 224. doi: 10.1186/s13059-019-1829-6 31661016 PMC6816165

[54] Gurevich A, Saveliev V, Vyahhi N, Tesler G. QUAST: quality assessment tool for genome assemblies. Bioinformatics. 2013;29: 1072–1075. doi: 10.1093/bioinformatics/btt086 23422339 PMC3624806

[55] Seppey M, Manni M, Zdobnov EM. BUSCO: assessing genome assembly and annotation completeness. In: Kollmar M, editor. Gene prediction: methods and protocols. New York, NY: Humana; 2019. pp. 227–245. 10.1007/978-1-4939-9173-0_14 31020564

[56] Li H, Durbin R. Fast and accurate short read alignment with Burrows-Wheeler transform. Bioinformatics. 2009;25: 1754–1760. doi: 10.1093/bioinformatics/btp324 19451168 PMC2705234

[57] Ramirez-Gonzalez RH, Bonnal R, Caccamo M, Maclean D. Bio-SAMtools: Ruby bindings for SAMtools, a library for accessing BAM files containing high-throughput sequence alignments. Source Code Biol Med. 2012;7: 6. doi: 10.1186/1751-0473-7-6 22640879 PMC3473260

[58] McKenna A, Hanna M, Banks E, Sivachenko A, Cibulskis K, Kernytsky A, et al. The Genome Analysis Toolkit: a MapReduce framework for analyzing next-generation DNA sequencing data. Genome Res. 2010;20: 1297–1303. doi: 10.1101/gr.107524.110 20644199 PMC2928508

[59] Clark SC, Egan R, Frazier PI, Wang Z. ALE: a generic assembly likelihood evaluation framework for assessing the accuracy of genome and metagenome assemblies. Bioinformatics. 2013;29: 435–443. doi: 10.1093/bioinformatics/bts723 23303509

[60] Lyčka M, Peška V, Demko M, Spyroglou I, Kilar A, Fajkus J, et al. WALTER: an easy way to online evaluate telomere lengths from terminal restriction fragment analysis. BMC Bioinformatics. 2021;22: 145. doi: 10.1186/s12859-021-04064-0 33752601 PMC7986547

[61] Duncan SM, Jones NG, Mottram JC. Recent advances in *Leishmania* reverse genetics: manipulating a manipulative parasite. Mol Biochem Parasitol. 2017;216: 30–38. doi: 10.1016/j.molbiopara.2017.06.005 28629934

[62] Boitz JM, Gilroy CA, Olenyik TD, Paradis D, Perdeh J, Dearman K, et al. Arginase is essential for survival of *Leishmania* donovani promastigotes but not intracellular amastigotes. Infect Immun. 2017;85: e00554–00516. doi: 10.1128/IAI.00554-16 27795357 PMC5203656

[63] Song K, Jung D, Jung Y, Lee SG, Lee I. Interaction of human Ku70 with TRF2. FEBS Lett. 2000;481: 81–85. doi: 10.1016/s0014-5793(00)01958-x 10984620

[64] Li B, Espinal A, Cross GA. Trypanosome telomeres are protected by a homologue of mammalian TRF2. Mol Cell Biol. 2005:25: 5011–5021. doi: 10.1128/MCB.25.12.5011-5021.2005 15923618 PMC1140576

[65] O’Connor MS, Safari A, Liu D, Qin J, Songyang Z. The human Rap1 protein complex and modulation of telomere length. J Biol Chem. 2004;279: 28585–28591. doi: 10.1074/jbc.M312913200 15100233

[66] Laffitte MN, Leprohon P, Papadopoulou B, Ouellette M. Plasticity of the *Leishmania* genome leading to gene copy number variations and drug resistance. F1000Res. 2016;5: 2350. doi: 10.12688/f1000research.9218.1 27703673 PMC5031125

[67] Sinha R, MM C, Raghwan, Das S, Das S, Shadab M, et al. Genome plasticity in cultured *Leishmania donovani*: comparison of early and late passages. Front Microbiol. 2018;9: 1279. doi: 10.3389/fmicb.2018.01279 30018594 PMC6037818

[68] Rogozin IB, Charyyeva A, Sidorenko IA, Babenko VN, Yurchenko V. Frequent recombination events in *Leishmania donovani*: mining population data. Pathogens. 2020;9: 572. doi: 10.3390/pathogens9070572 32679679 PMC7400496

[69] Kockler ZW, Comeron JM, Malkova A. A unified alternative telomere-lengthening pathway in yeast survivor cells. Mol Cell. 2021;81: 1816–1829. doi: 10.1016/j.molcel.2021.02.004 33639094 PMC8052312

[70] Bussotti G, Gouzelou E, Cortes Boite M, Kherachi I, Harrat Z, Eddaikra N, et al. *Leishmania* genome dynamics during environmental adaptation reveal strain-specific differences in gene copy number variation, karyotype instability, and telomeric amplification. mBio. 2018;9: e01399–01318. doi: 10.1128/mBio.01399-18 30401775 PMC6222132

