# Supplementary figures and images for: Ku80 is involved in telomere maintenance but dispensable for genomic stability in Leishmania mexicana
Source: PLoS Negl Trop Dis. 2021 Dec 29;15(12):e0010041. doi: 10.1371/journal.pntd.0010041 (PMC8716037; doi:10.1371/journal.pntd.0010041)

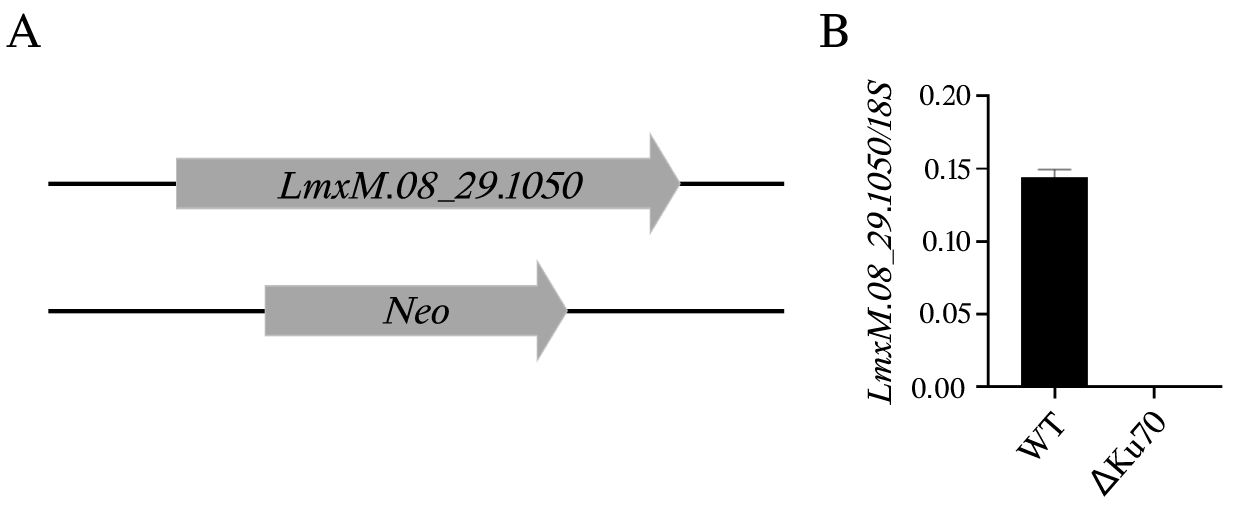

Supplement: S1 Fig — Establishment of the ΔKu70 L. mexicana. A) Strategy for LmxM.08_29.1050 ablation. B) Quantitative RT–PCR analysis of LmxM.08_29.1050 expression in WT and ΔKu70 L. mexicana strains. (JPG) [file pntd.0010041.s004.jpg]

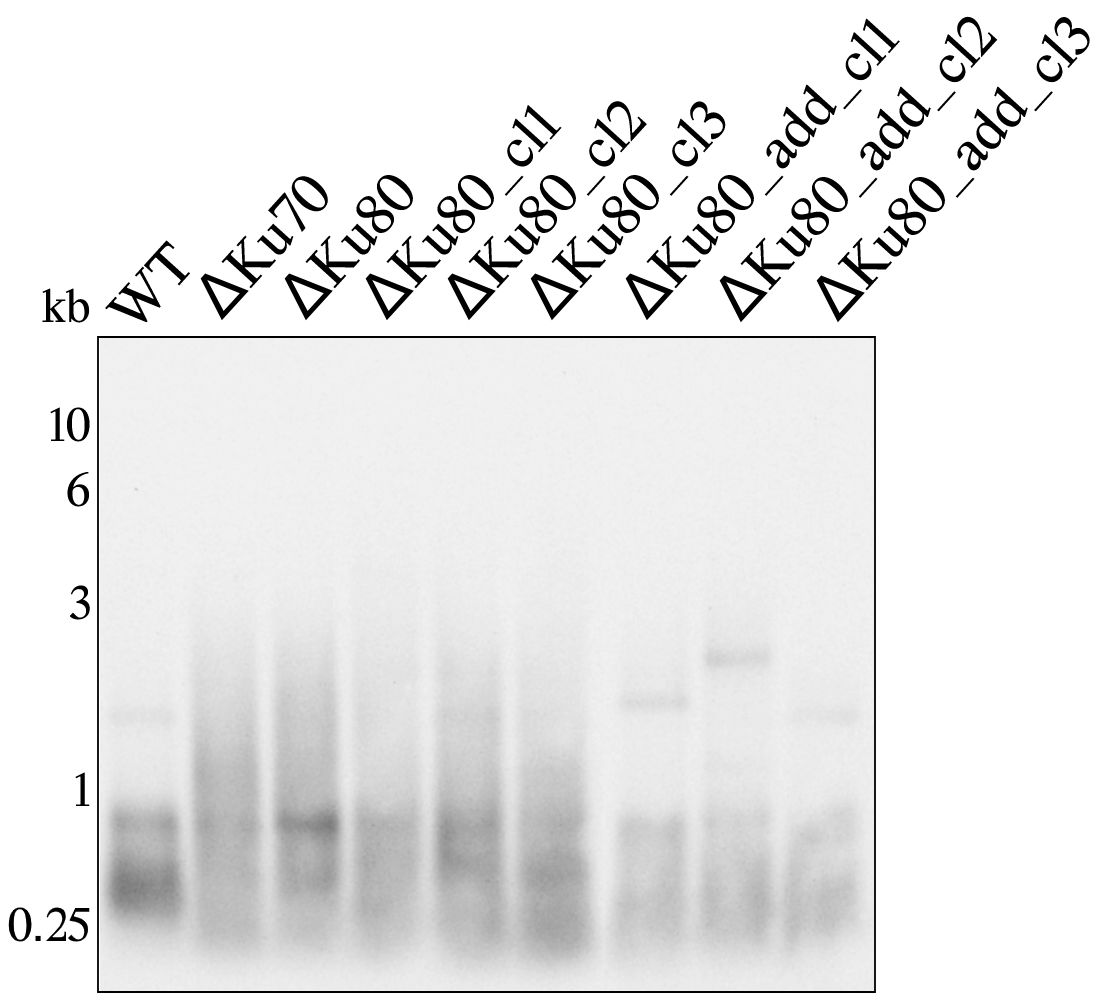

Supplement: S2 Fig — (JPG) [file pntd.0010041.s005.jpg]

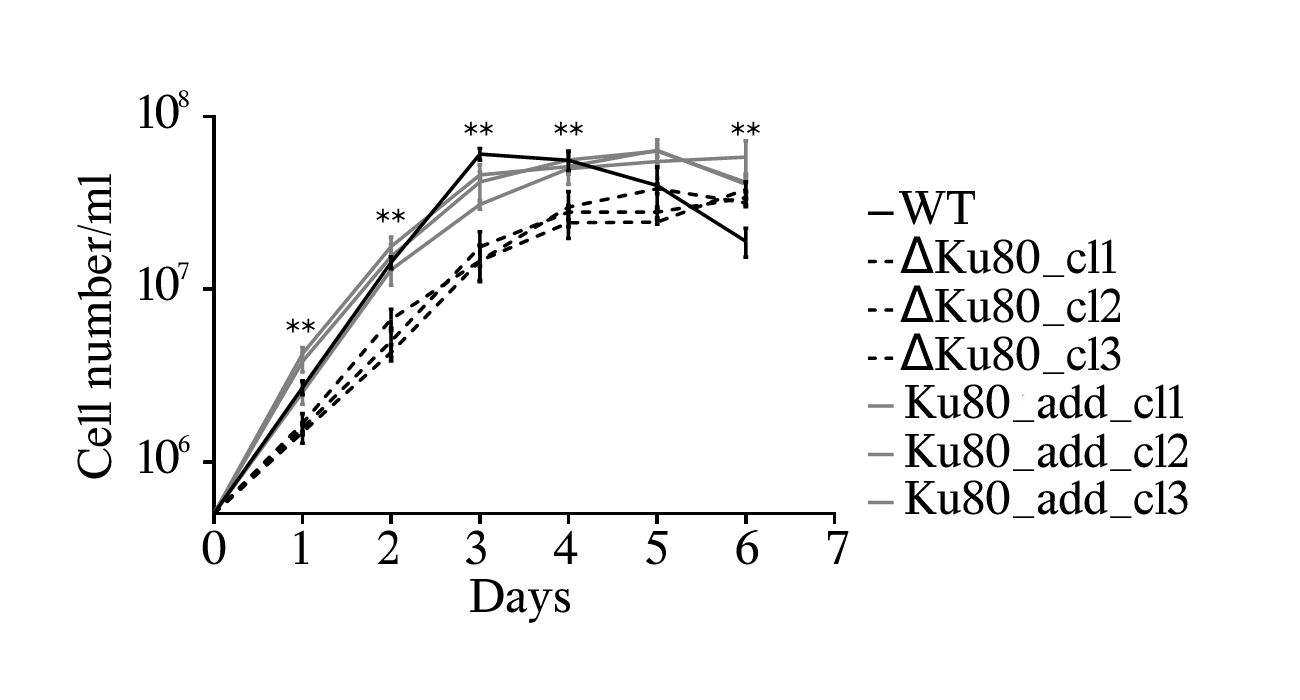

Supplement: S3 Fig — (JPG) [file pntd.0010041.s006.jpg]
